# University Organizational Support and Engineering Identity Among Engineering Postgraduates: The Sequential Mediating Role of Major Satisfaction and Engineering Belonging

**DOI:** 10.3390/bs16020199

**Published:** 2026-01-30

**Authors:** Weiwei Li, Jinfeng Lu, Liangyu Cao, Min Xiao

**Affiliations:** 1 Faculty of Education, East China Normal University, Shanghai 200062, China; 2 School of Education Science and Technology, Nanjing University of Posts and Telecommunications, Nanjing 210023, China; 3 Shaanxi Research Center for Academic Degrees and Graduate Education, Graduate School, Northwestern Polytechnical University, Xi’an 710072, China

**Keywords:** engineering postgraduates, university organizational support, major satisfaction, engineering belonging, engineering identity

## Abstract

Strengthening engineering identity among engineering postgraduates is essential to the sustainable development of the nation’s engineering talent teams. This subject has emerged as a major focus of international research on engineering education in recent years. A mediation model based on questionnaire data from 939 Chinese engineering postgraduates is employed to examine the relationship between university organizational support and engineering identity through the potential sequential mediators of major satisfaction and engineering belonging. The findings indicate that university organizational support is significantly correlated with engineering identity among postgraduates. University organizational support is statistically associated with engineering identity through independent and sequential mediation paths involving major satisfaction and engineering belonging. The results suggest that higher levels of university organizational support are positively correlated with greater major satisfaction and engineering belonging among engineering postgraduates, and these factors together are linked to stronger engineering identity.

## 1. Introduction

Engineering identity is, on average, considered a thorough reflection of engineering students’ comprehension and perception of their professional roles (Godwin, 2016). It is always evolving and encompasses a variety of dimensions of engineering students’ cognition, beliefs, emotions, and motivation toward professional roles. Engineering identity is central to engineering education research, with its importance widely acknowledged from multiple perspectives (Gómez et al., 2022). Research indicates that engineering identity is critically associated with the selection of engineering majors and retention (Doran & Swenson, 2022). Wallwey et al. (2024) found a substantial connection between engineering identity strength and students’ choice of engineering disciplines. After analyzing cross-sectional data from engineering undergraduates, A. D. Patrick et al. (2018) found that engineering identity is significantly associated with students’ continuous engagement in engineering programs. In a longitudinal study, Perez et al. (2014) demonstrated that students’ perception of their major’s value and long-term retention are positively correlated with engineering identity, which is developed through self-exploration. The relationship between engineering identity and career choice or development has also received widespread attention and discussion in academia. Morelock (2017) found that engineering identity is significantly linked to engineering students’ career options. Perkins et al. (2017) further indicated that engineering identity is positively related to doctoral students’ active participation in coursework, which is in turn associated with professional development. The stability of engineering identity is strongly correlated with important indicators of a nation’s engineering workforce stability and is essential to ensuring the quality of engineering education. A study emphasized that engineering identity serves as the foundation for long-term engineering major development, as well as being crucial to raising the quality of engineering education (Abdul Lahtif et al., 2025). Recent empirical research further suggests that students’ dedication to their careers, which is correlated with engineering identity, may contribute to the steady development of engineering education quality (Fatima & Ahmad, 2025).

The worldwide crisis of engineer shortage further emphasizes how important the engineering identity is. Many countries are currently facing serious challenges, such as insufficient engineering recruitment and a lack of talent. For example, according to a report released by the Association of German Engineers, there is a significant shortage of qualified technical personnel in Germany’s engineering sector, with engineering vacancies persistently elevated (Düsseldorf, 2024). About 35% of recently graduated engineers in the UK leave the engineering sector (Liquete et al., 2025). The dual pressure of persistent job vacancies and brain drain further intensifies the worldwide shortage of engineering talent. To counteract this challenge, governments have implemented targeted engineer education programs like Japan’s “Global Engineer Training Project”, and started emphasizing the training of future engineers. In light of this, developing engineering students’ engineering identities has arisen as a vital strategy for addressing the engineer shortage. Research indicates that engineering students’ future career choices and retention intentions are strongly associated with the engineering identities they develop during their higher education. A higher level of engineering identity is associated with the chance of choosing and pursuing an engineering career and over the long term, whereas a lower one is linked to intentions to leave the engineering field (Hughes et al., 2021; Lockhart et al., 2025). The group of engineering students, as the new force of the engineering team, urgently needs targeted support to shape their identity, which is crucial to ensuring the sustainable development of the national engineering sector.

Similar challenges, like the unattractive nature of engineering degree programs and the decline in graduate retention rates, appear in higher engineering education in China (F. Li et al., 2022). These real-world issues underscore the necessity of focusing on engineering students’ identity development. Notably, engineering identity exhibits significant variations across different educational stages (Godwin & Lee, 2017; Ju & Zhu, 2023). The postgraduate stage of engineering education in China, which is essential for engineering students to develop their professional identities, is the primary basis for this study’s focus on the engineering identities of engineering postgraduates. Engineering graduates typically spend at least two years of a three-year program on academic study and research training in Chinese education. However, there are not many chances for hands-on engineering practice and industrial contact. This training model suggests that the university’s internal educational environment plays a major role in the development of engineering post-graduates’ engineering identity (Yu & Ma, 2023). Therefore, this research identifies university organizational support as a key variable associated with engineering identity among engineering postgraduates. Existing research has preliminarily revealed that engineering postgraduates’ engineering identities and university organizational support are related. For example, Q. Chen et al. (2023) found that there was a strong correlation between engineering graduates’ university support and engineering identity. The formation of graduates’ engineering identity requires considering both external environmental support and individual psychological factors (Eliot & Turns, 2011). Among these, major satisfaction was defined as students’ feelings and attitudes toward professional learning and research tasks (Ding, 2019), which may be significantly correlated with engineering identity (Dehing et al., 2013; Liu et al., 2023). Furthermore, engineering belonging is recognized as another crucial psychological factor linked to engineering identity (Rohde et al., 2019; Jensen et al., 2023). Research in the past has explored the association between university organizational support, major satisfaction, engineering belonging, and the engineering identity of engineering students. However, the majority of research has examined the associations of these independent factors with engineering identity separately, with an emphasis on undergraduates. Current research lacks systematic integration of these variables to empirically test their sequential mediating relationships and their combined association with engineering identity among engineering postgraduates.

Therefore, this study aims to systematically explore the associations among university organizational support, major satisfaction, engineering belonging, and engineering identity among Chinese engineering postgraduates. Specifically, it proposes and tests a sequential mediation model in which major satisfaction and engineering belonging serve as successive mediators in the relationship between university organizational support and engineering identity. To this end, the research employs a questionnaire survey to collect data and conducts empirical analyses to examine the posited associations and mediation pathways. In doing so, it investigates the potential chained mediating roles of major satisfaction and engineering belonging. By addressing these questions, this study seeks to provide a contextualized examination of how university organizational support is linked to engineering identity within the distinctive context of Chinese postgraduate education. Furthermore, it aims to offer meaningful and practical insights for fostering a stronger engineering identity among engineering postgraduates in China.

## 2. Literature Review and Research Hypotheses

### 2.1. University Organizational Support and Engineering Identity

Social support theory served as the foundation for the concept of organizational support used in the study. “Organizational support” is one of the many categories into which social support can be classified based on its source and type. Organizational support in this study refers to both tangible and intangible support provided by the engineering postgraduates’ university, faculty, and other members within the institution during their academic training. Generally speaking, organizational support is thought of as comprising several components. A four-dimensional model consisting of instrumental, emotional, friendship, and informational support was put out by Cohen and Syme (1985). Consequently, Fang (2013) created a three-dimensional model of friendship support (instrumental, emotional, and informational support) by incorporating the aspects of friendship support relating to association and sense of belonging into the emotional support category. Instrumental support, such as financial aid and research equipment, is referred to as instrumental support. Emotional support includes closeness, respect, and interaction. Informational support includes practical help, like knowledge, advice, and guidance. This categorization enhances knowledge of organizational support and provides measurable standards for evaluating the support networks’ effectiveness within university organizational management.

The concept of “engineering identity” in engineering education has mainly been defined by scholars from three major viewpoints. The first one is the internal individual view. Jones et al. (2014) defined the engineering identity of students as the subjective cognition of their own engineering roles and performance. The second perspective, which is about engineering students’ community belonging, is the external classification perspective (Major & Kirn, 2017). The third perspective is all-encompassing and views engineering identity as the alignment between students’ self-identity and the recognition they receive from others (Perkins et al., 2017). In general, engineering identity formation is a dynamic process that involves continuous reflection and adjustment, shaped by social interactions and personal psychological characteristics. As a multidimensional construct, it is associated with numerous factors, including internal psychological traits such as learning motivation (Y. Li & Singh, 2022), self-belief (Hughes et al., 2021; Eliot & Turns, 2011), and personal capabilities (Schell et al., 2021). Moreover, it is influenced by external factors, involving campus atmosphere (Sanchez et al., 2024; Bahnson et al., 2023), practical experience (Choe et al., 2019), interpersonal interactions (Ju & Zhu, 2023), and sociocultural and institutional factors (J. Chen et al., 2023). Different focuses are also evident in the engineering identity measurement tools. The study concentrates on the overall engineering identity and uses a single or a few items to assess how much students regard themselves as engineers (Borrego et al., 2018; Meyers et al., 2012). This measurement method typically falls into the “minority”. Another study regards engineering identity as a multifaceted latent variable. For example, an engineering identity formation scale comprising three dimensions—engineering commitment, exploration depth, and reflection on commitment—was created by N. Li et al. (2021). Engineering interest, engineering cognition, engineering ability, and interpersonal skills are the four dimensions of engineering identity, according to Choe and Borrego (2019). This study primarily adopts the three-dimensional evaluation framework proposed by Godwin and Kirn (2020), which includes cognition, interest, and performance/ability belief, to assess the students’ engineering identity. Strong reliability and validity have been demonstrated by this framework, which has passed the systematic psychometric test (Lockhart et al., 2025), and matches the environment of postgraduate engineering education in China. It is currently acknowledged as an effective standardized measurement tool.

The continuous interaction between external situations and internal cognition plays a key role in the construction of engineering identity (Eliot & Turns, 2011), a process that can be explained by social cognitive theory. This theory emphasizes how individual activities are shaped by the dynamic interaction of personal, behavioral, and environmental factors. Within this framework, the support provided by the external environment is regarded as a crucial contextual factor associated with individual cognitive and identity development. Multiple studies have demonstrated a significant association between university organizational support and engineering identity (Garriott et al., 2019). For example, a Danish study found that external organizational support was significantly correlated with the construction of students’ engineering identity (J. Chen et al., 2023). Sanchez et al. (2024) reported that professional development opportunities and experiences offered by university engineering student organizations are positively correlated with students’ engineering identity. Students’ professional identities and departmental emotional support were found to be somewhat positively correlated in an empirical study by Jensen and Cross (2021). Based on a sample of 522 students from Chinese universities, Yu and Ma (2023) identified a substantial positive correlation between institutional support and professional identity among engineering students. Although the relationship has not been thoroughly investigated specifically among postgraduates, existing evidence suggests that higher levels of university organizational support are linked to postgraduates’ access to resources that meet academic and research needs, which in turn correlates with their self-efficacy and sense of professional belonging in research and practice (Bahnson et al., 2023). The study puts forward the following hypothesis, building on these theoretical foundations:

**H1.**  *University organizational support is positively associated with engineering identity among engineering postgraduates.*

### 2.2. The Mediating Role of Major Satisfaction

Students’ degree of satisfaction with their chosen subject is referred to as major satisfaction (Nauta, 2007). As a psychological feeling formed by students in the process of professional learning, it illustrates the difference between students’ professional expectations and actual experiences (Simmons et al., 2017). Major satisfaction in engineering education involves students’ evaluation of curricula, teaching resources, and developmental support. This variable was closely linked to the formation of their engineering identity and career choice. Recently, research has increasingly highlighted the inherent connection between major satisfaction and engineering identity. Numerous studies have revealed that major satisfaction and engineering identity among engineering students are significantly correlated (Godwin & Kirn, 2020; Burleson et al., 2021; Flores et al., 2021). Liu et al. (2023) adopted the structural equation model to further verify this association. Vogel and Human-Vogel (2018) found that major satisfaction is linked to engineering students’ academic engagement and engineering identity in terms of the mechanism of action. The positive association between major satisfaction and the careers of engineering students was confirmed by further research (Fouad et al., 2011), demonstrating substantial connections among major satisfaction, career choice, and engineering identity.

A large amount of theoretical research has profoundly investigated the relationship between organizational support and major satisfaction. The organizational support theory deems that individuals are likely to form positive emotional and cognitive evaluations when they believe their organization values their contributions and cares about their development (Eisenberger et al., 1986; Han et al., 2023). It has been demonstrated that students’ perceptions of university support are significantly positively correlated with their satisfaction with teaching resources and training environment in the context of higher education (Trullàs et al., 2018). For instance, Soria and Stebleton (2013) found that high-quality interactions between teachers, students, and peers are positively connected with students’ (particularly working-class university students) satisfaction with their majors. Meanwhile, research by Yang (2023), based on data from 245 Chinese university students, suggested that university organizational support is linked to major satisfaction through learning engagement. Q. Chen et al. (2023) further noted that high-quality teacher support and favorable learning settings facilitate students’ positive major evaluations, which, in turn, correlate positively with their major satisfaction. Furthermore, research by Rohde et al. (2019) found that engineering students’ overall satisfaction with engineering programs is associated with supportive university environments that effectively alleviate their ambiguity regarding academic pressure and professional identity. Based on the above theoretical and empirical analysis, this study implies that university organizational support is associated with major satisfaction, which in turn is linked to engineering identity among engineering postgraduates. Specifically, the perception of stronger university organizational support may correlate with higher major satisfaction, and this heightened satisfaction may be associated with a stronger sense of engineering identity. Therefore, this study proposes the following hypothesis:

**H2.**  *Major satisfaction may mediate the relationship between university organizational support and the engineering postgraduates’ engineering identities.*

### 2.3. The Mediating Role of Engineering Belonging

Engineering belonging refers to engineering students’ subjective sense of acceptance, adaptation, and inclusion within their major. It is explicitly reflected in their perception of being valued and their emotional connection as legitimate members of the engineering community (Simmons et al., 2019). Substantial studies have indicated that engineering belonging significantly correlates with the engineering students’ identities (Jones et al., 2013; Rodriguez et al., 2018; Marra et al., 2012). Students with lower levels of engineering belonging are prone to feel alienated and confused about their professional identity, as well as lack clear professional goals and development motivation. Instead, students with a stronger sense of engineering belonging are more likely to report actively addressing academic challenges and internalizing the norms and values of the engineering discipline, factors that are associated with a more stable and positive professional self-concept. Fletcher et al. (2023) further emphasized the protective role of engineering belonging for the identity construction of marginalized student groups. Additionally, Tonso (2014) noted that the engineering belonging is essential to preserving the stability of engineering students’ identities. A. Patrick et al. (2023) emphasized that engineering identity varies in relation to the strength of students’ sense of belonging within their communities. Ong et al. (2020) also found that engineering belonging is positively correlated with students’ academic persistence and career aspirations, further supporting its close link to engineering identity.

One important contextual factor closely associated with the development of engineering students’ belonging is university organizational support. Polmear et al. (2024) carried out an empirical survey of 849 engineering undergraduates from six universities. They found that positive interactions with students, peers, and faculty are significantly correlated with students’ sense of belonging. From a sociocultural standpoint, students’ sense of belonging is closely linked to the department environment, which serves as a space for interaction between dominant and non-dominant groups (Davis et al., 2023). Litzler and Samuelson (2013) further confirmed that there is a strong correlation between engineering belonging, extracurricular participation, and teacher-student relationships. Research has also demonstrated a favorable correlation between the level of organizational support provided by universities and engineering students’ belonging (Garriott et al., 2019; Tate & Linn, 2005). Students who perceived stronger organizational support usually reported greater integration into professional communities, higher efficacy in coping with academic challenges, and stronger levels of engineering identity. Based on the aforementioned theoretical framework and empirical research, the study holds that university organizational support is not only directly associated with engineering belonging but may also be indirectly linked to engineering identity through this sense of belonging. Therefore, the following hypothesis is put forward:

**H3.**  *Engineering belonging mediates the relationship between university organizational support and engineering identity among engineering postgraduates.*

### 2.4. The Sequential Mediating Role of Major Satisfaction and Engineering Belonging

The research above highlights the importance of intrinsic psychological perceptions and emotional connections in linking the external environment to identity formation. Accordingly, the primary aim of the study is to explore how major satisfaction and engineering belonging sequentially mediate the relationship between university organizational support and engineering identity among engineering postgraduates, which has a solid theoretical foundation. The theoretical framework is grounded in social cognitive career theory (Lent & Brown, 2013), which integrates key principles from self-efficacy and broader social cognitive theories. This theory emphasizes that career development is influenced by the complex dynamic interaction of personal characteristics, external environmental influences, and behavioral performance. From this perspective, individuals’ professional identity is directly linked to their external environment, which also indirectly shapes their learning experience and emotional connection (Lent et al., 2000; Park et al., 2023). In particular, engineering postgraduates can enjoy active professional participation through high-level university support. These experiences, internalized as satisfaction evaluations of their academic studies, shape their cognitive and emotional responses to professional activities. A deeper and more stable sense of belonging—that is, the identification and emotional attachment to the engineering community and its value culture—may develop over time from cumulative satisfaction. Ultimately, a stronger engineering identity is associated with the combination of cognitive satisfaction with professional activities and emotional identification as a member of the engineering community. Therefore, major satisfaction and engineering belonging may function as sequential mediators between organizational support and engineering identity, representing a progression from cognitive evaluation to emotional attachment, according to the social cognitive career theory. Indeed, preliminary evidence for the mediating role of major satisfaction and engineering belonging has been provided in existing empirical research. For example, Polmear et al. (2024) found that major satisfaction mediated the relationship between peer and faculty interactions with engineering belonging in the surveys of 849 engineering undergraduates across six universities. Furthermore, major satisfaction and engineering belonging are strongly positively correlated, according to Fan et al.’s (2020) empirical research, with belonging being essential in connecting learning experiences to professional identity.

**Figure 1:**
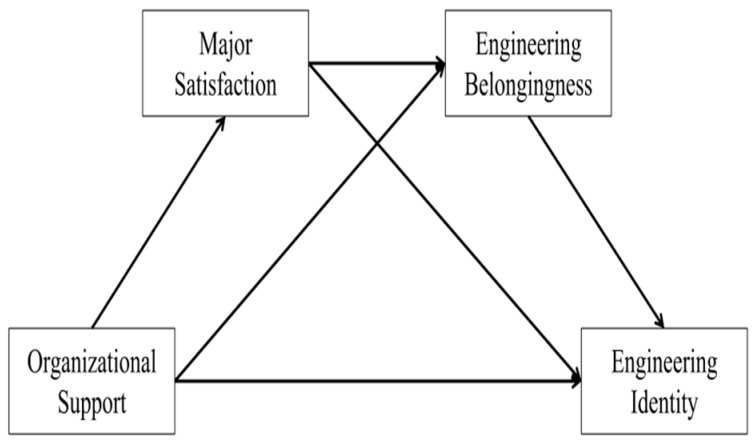
Hypothesized research model.

However, a comprehensive review of the current literature identifies several areas that warrant further exploration in this field. First, the specific pathways and mechanisms through which major satisfaction and engineering belonging link organizational support to engineering identity remain unclear and have not been explicitly defined in the context of postgraduate education. Second, few studies have focused specifically on Chinese engineering postgraduates, whose identity formation may be influenced by distinct contextual factors. The intricate associations between organizational support and engineering identity in the Chinese context have not been fully examined, thereby limiting the development of models that reflect the psychological patterns of this population. Therefore, this study focuses on Chinese engineering postgraduates. Drawing on social cognitive career theory, we propose a chained mediation model (Figure 1) in which major satisfaction and engineering belonging serve as sequential mediators in the relationship between university organizational support and engineering identity. Thus, the following hypothesis is proposed:

**H4.**  *Major satisfaction and engineering belonging sequentially mediate the relationship between university organizational support and engineering identity among engineering postgraduates.*

The theoretical hypothesis framework is diagrammatically shown in Figure 1, which elucidates the interrelationships among the variables in the proposed model.

## 3. Research Methods

### 3.1. Research Sample and Data Sources

The research data was collected via a questionnaire survey, which was made by the research team in the form of an electronic link through the Questionnaire Star platform. Supported by the graduate school managers of relevant institutions, the questionnaire was distributed to WeChat groups, QQ groups, and via email, lasting for nearly two months and recovering a total of 1084 questionnaires. Approximately 145 invalid questionnaires were excluded with an initial review, and 939 valid questionnaires were retrieved, resulting in a feedback rate of 86.62%. According to Kline’s (1998) minimum sample size for structural equation modeling (10 times the number of questionnaire items), the minimum sample size required for the 34 questionnaire items involved in this study is 340, indicating that the final sample meets the statistical requirements. It is necessary to conduct a variance analysis to avoid significant differences in sample collection methods. The results revealed no statistically significant differences among the samples, proving the suitability for further data analysis.

The demographic data of the participants in Table 1 pinpointed that male participants accounted for 59.96%, and female participants were 40.04%. Regarding degree plane, those with master’s degrees accounted for 64.11%, while those with doctorates made up 35.89%. As for university level, the distribution of non-double first-class universities was 38.13%, and the percentage of double first-class ones was 61.87%. In terms of academic performance, the proportions of participants who scored below 20%, 20–40%, 40–60%, 60–80%, and above 80% were 12.35%, 19.70%, 36.21%, 21.73%, and 10.01%, respectively. Additionally, 64.11% of the participants were engineering postgraduates without practical experience, compared to 35.89% graduates.

### 3.2. Scale Revision and Validation

The study methodically improved the scales to guarantee the cultural relevance, content clarity, and psychometric robustness of the measurement instruments for Chinese engineering postgraduates. An extensive search for validated scales relevant to the research variables was conducted initially, and the selected scales were systematically translated and back-translated. Therefore, two rounds of content validity assessments were launched. Five experts in higher engineering education participated in the first round. They assessed the accuracy of each item in measuring the desired construct while being mindful of its theoretical significance and domain suitability. The second round included eight engineering postgraduates, who evaluated the items for semantic clarity and contextual understanding. Targeted revisions were made to the items according to the feedback. For example, “I am satisfied with my chosen major” was revised to “Overall, I am satisfied with my selected engineering major” for a better reflection of Chinese postgraduates’ experiences. This study has developed a preliminary questionnaire for pretesting, which involved modifying the wording of 9 items and eliminating 2 items with cultural bias and understanding barriers.

This study performed a preliminary survey with 174 randomly selected engineering postgraduates who were chosen at random in September 2025. Reliability tests revealed that all four dimensions had Cronbach’s alpha coefficients exceeding 0.75, which is acceptable. Validity analysis indicated a KMO value of 0.894 and Bartlett’s test chi-square value of 2408.12 (*p* < 0.001), confirming the data’s suitability for exploratory factor analysis. Three items that failed to match the requirements of factor loadings ≥0.50 and no significant cross-loadings were eliminated. Finally, it revealed a clear four-factor structure, explaining 68.7% of the variance. Factor loadings for all retained items ranged from 0.52 to 0.74, which was in line with the original theoretical dimensions.

### 3.3. Measurement Tools

#### 3.3.1. University Organizational Support Scale

An instrument developed by Zhou et al. (2019) and adapted to the unique demands of engineering graduate education was used in the University Organizational Support Scale. The scale consists of 10 items categorized into three dimensions: instrumental support, emotional support, and informational support. Example items include: “The university or school provides reasonable financial support for postgraduates, such as scholarships and grants”. The scale was evaluated through a 5-point Likert scale; the higher the score, the greater the organizational support from the university was received. The Cronbach’s alpha coefficient for the scale was 0.882. The confirmatory factor analysis yielded the following results: X^2^/df = 1.112, RMSEA = 0.011, SRMR = 0.015, CFI = 0.999, and NFI = 0.989. The scale demonstrates good fit indices, which may be mainly due to the study’s large sample size and its clear structure after systematic revisions. Only two semantically similar items in the “emotional support” dimension could have error term correlation during model fitting. It is justified in theory and does not affect the three-factor structure. The analysis verifies that the scale’s validity falls within a reasonable range.

#### 3.3.2. Major Satisfaction Scale

To measure the major satisfaction of engineering postgraduates, the study applied five items developed by Simmons et al. (2019). For example, “I am enthusiastic about the major I have chosen”. A 5-point Likert scale was employed to assess these items, with higher scores indicating greater agreement. Meanwhile, the Cronbach’s alpha coefficient of the scale was 0.74. The confirmatory factor analysis results were as follows: X^2^/df = 1.635, RMSEA = 0.026, SRMR = 0.015, CFI = 0.996, and NFI = 0.991, which suggested that the scale has acceptable reliability and validity.

#### 3.3.3. Engineering Belonging Scale

The engineering belonging scale was devised based on the General Sense of Belonging Scale (GBS) (Malone et al., 2012). It was modified to pinpoint the experiences of Chinese engineering postgraduates. There are six items in this scale with sample items including “I feel recognized by the teachers and classmates in my class”. The items were rated through a 5-point Likert scale, with the higher scores signifying stronger engineering belonging. The Cronbach’s alpha coefficient for the scale was 0.782, indicating acceptable reliability. The confirmatory factor analysis yielded the following results: X^2^/df = 1.808, RMSEA = 0.029, SRMR = 0.018, CFI = 0.994, and NFI = 0.987.

#### 3.3.4. Engineering Identity Scale

This research used the engineering identity scales developed by Godwin and Kirn (2020) and Choe and Borrego (2019), with modifications and improvements made to suit the particular circumstances. As a result, a 13-item scale was developed to assess the engineering identity of engineering postgraduates, with three dimensions of engineering recognition, engineering interest, and engineering belief. Example items include “My tutor regards me as an engineer”. The items are evaluated via a 5-point Likert scale, with higher scores signifying stronger engineering identity, and the Cronbach’s alpha coefficient was 0.914. The results of the confirmatory factor analysis were as follows: χ^2^/df = 0.819, RMSEA = 0.01, SRMR = 0.013, CFI = 0.999, and NFI = 0.99. The sample’s homogeneity and the scale’s rigorous revision procedure are probably responsible for the scale’s excellent fit indices. During model fitting, error term correlations were allowed between two items related to actual participation in the “engineering interest” dimension to reflect their common methodological characteristics, without introducing cross-factor correlations. Consequently, a conclusion is drawn that the scale exhibits strong reliability and validity.

### 3.4. Statistical Analysis

The study employed Amos 26.0 and SPSS 27.0 to analyze the data. Among these, the measurement model’s internal consistency and construct validity were assessed using Amos 26.0. Moreover, descriptive statistics were conducted to explore the overall levels of perceived university organizational support, major satisfaction, engineering belonging, and engineering identity among engineering postgraduates, as well as the correlations among these variables. While PROCESS v4.0 was applied to test a sequential mediation model, examining the relationship between university organizational support and engineering identity through the mediating effects of major satisfaction and engineering belonging.

## 4. Results

### 4.1. Common Method Bias Test

Both Harman’s single-factor approach and the unmeasured latent construct approach were used to assess common method bias concurrently (Podsakoff et al., 2003). According to the Harman test, the initial common factor accounted for 27.875% of the variance, which is below the critical threshold of 40%. The confirmatory factor analysis models M1 and M2 were thus developed for further examination, each with a latent method factor. A comparison of the main fit indices between these two models demonstrated: ΔCFI = 0.009, ΔNFI = 0.014, ΔRMSEA = 0.014, ΔIFI = 0.009, and ΔSRMR = 0.026. None of the model fit indices exceeded 0.03, indicating that the inclusion in the common method factor did not substantially improve the model fit. Overall, these results suggest that although common method bias cannot be completely eradicated, it does not seriously jeopardize the core conclusions of this study.

### 4.2. Descriptive Statistics and Correlation Analysis

The descriptive statistics and correlation analysis results for university organizational support, major satisfaction, engineering belonging, and engineering identity are shown in Table 2. The mean scores for all four variables were near the midpoint of the 5-point Likert scale. In particular, university organizational support averaged 3.09 (SD = 0.68), major satisfaction 3.15 (SD = 0.63), engineering belonging 3.18 (SD = 0.65), and engineering identity 3.19 (SD = 0.69). The minimal variation between these means indicates that no single construct was markedly higher or lower than the others. On the whole, university organizational support, major satisfaction, engineering belonging, and engineering identity all remain at a moderately low level, categorized as “barely acceptable”. Among these variables, university organizational support enjoyed the lowest mean score, which suggests that current institutions of higher education can still have room to improve the educational environment support.

The Pearson correlation analysis confirmed that the university organizational sup-port significantly correlated with major satisfaction (r = 0.495, *p* < 0.01), engineering belonging (r = 0.405, *p* < 0.01), and engineering identity (r = 0.327, *p* < 0.01). Major satisfaction indicated significant positive correlations with engineering belonging (r = 0.431, *p* < 0.01) and engineering identity (r = 0.403, *p* < 0.01). Moreover, there was a significant positive relationship between engineering belonging and engineering identity (r = 0.405, *p* < 0.01). The aforementioned correlation results offer a preliminary foundation for subsequently building and evaluating a mediation model among variables to examine their potential associative pathways.

### 4.3. Mediation Model Test

**Figure 2:**
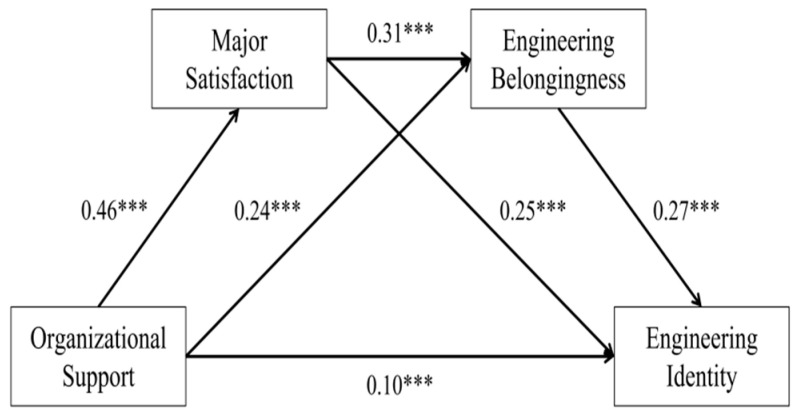
The sequential mediating model (Note: *** indicates *p* < 0.001).

Model 6 from the PROCESS macro developed by Hayes (2012) was used in this study. The research investigated the mediating effects of major satisfaction and engineering belonging on the relationship between university organizational support and engineering identity after controlling for gender, degree level, school type, academic ranking, and engineering practice experience. The bias-corrected percentile bootstrap method was used in the analysis, and the results were evaluated using 95% bias-corrected confidence intervals (CI). A total of 5000 random samples were repeatedly drawn from the initial dataset. A statistically significant mediating effect is shown by a non-zero CI (Preacher & Hayes, 2008). The R^2^ value of 0.1313 indicates that the chained mediation model explained 13.13% of the variance in the engineering identity variable, as demonstrated in Table 3 and Table 4 and Figure 2. The F-statistic was 23.481, with a highly significant *p*-value of 0.000. The proposed chained mediation model is supported by the data and helps to comprehend the statistical relationships among variables, according to the overall model’s statistical significance.

Regression analysis (see Table 3 and Figure 2) revealed significant positive correlations between university organizational support, major satisfaction (β = 0.46, t = 17.19, *p* < 0.001), and engineering belonging (β = 0.24, t = 7.70, *p* < 0.001). University organizational support also showed a significant positive association with engineering identity (β = 0.10, t = 2.39, *p* < 0.001), supporting Hypothesis 1. Both engineering belonging (β = 0.31, t = 9.00, *p* < 0.001) and engineering identity (β = 0.25, t = 6.76, *p* < 0.001) were significantly positively correlated with major satisfaction. Furthermore, engineering belonging was significantly positively associated with engineering identity (β = 0.27, t = 7.83, *p* < 0.001).

The sequential mediating analysis (Table 4) revealed that university organizational support had a substantial overall indirect effect on engineering identity (effect = 0.220), accounting for 68.59% of the total effect.

This overall mediation comprised three distinct pathways:

(1) University organizational support → major satisfaction → engineering identity. The indirect effect of this pathway is 0.116, making up 36.13% of the total indirect effect. Its 95% confidence interval (CI) is [0.080, 0.155], excluding zero, thereby providing support for Hypothesis 2.
(2) University organizational support → engineering belonging → engineering identity. The indirect effect for this pathway is 0.066, with 20.63% of the total indirect effect. Its 95% CI is [0.044, 0.093], which does not include zero, supporting Hypothesis 3.
(3) University organizational support → major satisfaction → engineering belonging → engineering identity. This pathway’s indirect effect is 0.038, about 11.84% of the total indirect effect. Its 95% confidence interval of [0.025, 0.053] excludes zero, confirming the statistical significance of this pathway and supporting Hypothesis 4.

## 5. Discussion

### 5.1. University Organizational Support and Engineering Postgraduates’ Engineering Identity

The study finds a significant positive correlation between university organizational support and engineering identity among postgraduates. This finding holds deeper significance within China’s highly structured engineering graduate education system, even though it is compatible with current theories. In Chinese institutions, organizational support is crucial for students’ identity construction since it emerges as a source of institutional recognition and identity legitimacy and provides resources. The three dimensions of support—instrumental, emotional, and informational—were all closely linked to engineering identity, together forming important associated factors in its development. First, universities are compelled to give instrumental support, such as funding and research equipment, by national initiatives like the “Double First-Class” program, which establishes the material foundation for students to engage in engineering practice and scientific innovation (Lachance et al., 2020). Sufficient resources are associated with students’ perceptions of a “superior platform,” which in turn is linked to higher self-efficacy in engineering tasks and greater internalization of professional roles (Lent et al., 2007). Second, emotional support from universities and departments is particularly vital given the competitive and demanding nature of graduate education in China. Such support not only alleviates anxiety and isolation but is also associated with an enhanced sense of belonging within the engineering community and the formation of a more stable professional identity during students’ exploratory phases (Wang et al., 2025; Lee et al., 2025). Moreover, informational support, conveyed through policy communication, academic exchanges, and career resource sharing, helps shape students’ understanding of engineering career pathways and their perceptions of future professional roles (Shen et al., 2024). Therefore, within highly competitive and organized educational environments like China’s, systematic university organizational support is associated with perceptions of legitimacy, emotional security, and clarity regarding developmental trajectories.

### 5.2. The Mediating Role of Major Satisfaction

The association between university organizational support and engineering identity is substantially mediated by major satisfaction. The discrepancy between expectations and actual experiences affects an individual’s satisfaction according to customer satisfaction theory (Oliver, 1980). Expectations for Chinese engineering postgraduates are shaped by their own interests, cultural pressures from their family and societal expectations, and unspoken responsibilities related to university identity. Their “perceived experiences” are incorporated into an institutionalized training program that includes an advisor responsibility system, rigorous midterm assessments, and graduation requirements. University organizational support is associated with more positive evaluations of professional training experiences, which may reflect a reduced discrepancy between students’ expectations and perceived academic outcomes. Accordingly, this correlates with increased satisfaction within their discipline. Major satisfaction, reflecting students’ evaluation of the value of engineering, the training system, and career prospects (Pablo-Lerchundi et al., 2015), is closely associated with their sense of career fit and professional dedication. In China’s competitive educational environment, engineering postgraduates who are more satisfied with their majors are more likely to incorporate the values, ethics, and mindset of the engineering discipline into their self-concept. A heightened sense of identification with the engineer identity is correlated with this internalization process. On the contrary, students may feel disconnected from the fundamental principles of the engineering profession and doubt their potential as qualified engineers if major satisfaction stays low. Such perceptions are associated with challenges in developing a robust engineering identity and may correlate with its weakening or with intentions to pursue alternative career paths.

### 5.3. The Mediating Role of Engineering Belonging

This study finds that engineering belonging serves as a significant mediator in the association between university organizational support and engineering identity. Throughout their academic careers, engineering postgraduates encounter a diversity of challenges. High levels of university organizational support are linked to long-term academic progress and help students acquire essential professional skills that form the foundation of their engineering identity. The research further supports the findings of prior research (Quezada-Espinoza & Truyol, 2024). They show that engineering postgraduates who receive sufficient university or organizational support exhibit higher levels of engineering belonging. Students can more easily become part of the “engineering community” that is formed through faculty mentorship, research teams, and industry-based projects in a supportive academic environment. A strong sense of emotional belonging is closely tied to students’ recognition of their value as members of this group. In Chinese culture, which emphasizes collective identity and professional belonging, strong engineering belonging not only provides emotional support but is also directly associated with a stronger professional identity (Antonio & Baek, 2022). When confronted with rigorous research training and engineering challenges, students with a stronger sense of belonging show greater psychological resilience and engage more actively in a variety of engineering practices. This active engagement is associated with higher levels of reported engineering identity. Furthermore, among Chinese engineering postgraduates, this sense of belonging is linked to alleviating the mental strain and anxiety brought on by research training (Miller & Orsillo, 2020), stabilizing their professional development. In contrast, engineering postgraduates who lack effective links with the engineering community during their training and struggle to acquire recognition and acceptance from academic teams or industrial groups are more likely to experience professional alienation. According to an earlier study, this enables people to question their suitability for the engineering profession and is associated with obstacles to the development of their engineering identity (Danielak et al., 2014).

### 5.4. The Sequential Mediating Role of Major Satisfaction and Engineering Belonging

The findings indicate that university organizational support is associated with engineering identity among engineering postgraduates through a sequential mediating pathway involving major satisfaction and engineering belonging. Specifically, higher perceived university organizational support is linked to greater major satisfaction among engineering postgraduates. This positive evaluation of their professional experience is, in turn, associated with a stronger sense of emotional belonging within the engineering domain, which correlates with higher levels of engineering identity. Identity formation theory can be used to analyze this sequential pathway. This theory emphasizes that a person’s identification with a particular social role is a gradual process of internalization (Ye et al., 2025), usually starting with favorable emotional experiences connected to role activities and ending with the establishment of group belonging (Eccles, 2009). In this study, higher major satisfaction, stemming from university-provided resources that meet research and practical needs, is associated with students’ active integration into engineering practice communities. Through interactions with peers, supervisors, and industry experts, they gradually develop a sense of group belonging based on a shared “we engineer” identity (Burleson et al., 2021; Hatmaker, 2013). This sense of belonging is closely linked to the incorporation of the engineer role into one’s self-concept (Verdín, 2021). Major satisfaction and engineering belonging therefore represent statistically significant intermediary constructs linking university organizational support and engineering identity. This mechanism also suggests that there may be a weakening of the positive association between university organizational support and heightened engineering identity within the Chinese context if it is unable to effectively correlate with enhanced student major satisfaction or deeper connections to their engineering community. It should be mentioned that the model only accounts for 13.13% of the variance in engineering identity, despite the sequential mediating pathway found in this study being statistically significant. This suggests that the tested pathway represents one important component within a more complex formation mechanism. Consequently, care should be taken when interpreting and generalizing the results of this research to prevent overgeneralization.

### 5.5. Theoretical Implications

This study offers contextual theoretical contributions by examining established identity-related pathways within Chinese postgraduate engineering education. First, it extends the understanding of professional identity formation by examining the association between university organizational support and engineering identity among postgraduates. By analyzing the sequential mediating roles of major satisfaction and engineering belonging, the study tests the robustness of an established psychological pathway in the context of Chinese engineering postgraduates. Second, it integrates macro-organizational environmental factors into the established theoretical pathway linking satisfaction, belonging, and identity, thereby testing and supporting the robustness of this pathway in a distinct context. This highlights the important role of institutional support in identity formation and encourages the development of more comprehensive, multi-level analytical frameworks. Finally, the study validates this established model within the unique setting of Chinese postgraduate education, characterized by the advisor-responsibility system and a highly competitive academic environment. This work enhances the cross-cultural applicability of relevant theories and provides important empirical evidence for understanding how specific institutional and cultural contexts shape professional identity, thereby contributing to both global discourse and contextual adaptation in the field.

### 5.6. Practical Implications

There are three practical insights from this study for systematically improving engineering identity among engineering postgraduates in higher education institutions. On the one hand, universities should strengthen their organizational support systems to create a supportive educational environment. This calls for the certainty of adequate research resources and practical conditions, such as establishing shared equipment platforms and diverse funding mechanisms to make students feel more institutional support. Moreover, a greater premium should be placed on emotional support. Interventions, including ongoing mentorship, psychological consultancy, and peer academic companionship, can help alleviate academic pressure and identity anxiety, which may contribute to a stronger sense of belonging and engineering identity (Jensen et al., 2023; Bork & Mondisa, 2022). Furthermore, improving information support mechanisms is essential to effectively disseminate key information on training policies, research opportunities, and career development. Such transparency empowers students to make wise decisions and enhances their satisfaction with university support.

On the other hand, focusing on and improving the major satisfaction of engineering postgraduates represents a key lever for strengthening their engineering identity. Universities should optimize their curriculum systems, make a balance between academic and applied components, strengthen the integration of engineering case teaching and interdisciplinary approaches, and guide students to deepen their professional cognition and practical abilities in solving real-world problems (Wei et al., 2025). In terms of mentorship, they should allow teachers and students to interact more, provide tailored support and positive feedback, and motivate students to develop a favorable assessment and maintain continuous interest in their majors (Hund et al., 2018). Higher education institutions should simultaneously build a multidimensional evaluation mechanism to encourage students to achieve a variety of accomplishments in scientific research and innovation, engineering practice, and social services, thereby improving their overall major satisfaction.

Additionally, to strengthen engineering postgraduates’ sense of belonging to support stable identity formation. Colleges and universities should promote education in campus-enterprise collaboration and establish joint laboratories and engineering practice bases to permit students to experience their own roles and values in real-world engineering scenarios (Shah & Gillen, 2024). Peer cooperation and collective recognition are meant to be enhanced through scientific research teams, innovation groups, and other forms of learning communities, in addition to strengthening the guidance on values and identity formation. Students’ knowledge of the social responsibilities and contemporary missions of engineers should be deepened through activities like highlighting exemplary engineers and organizing engineering culture forums.

## 6. Limitations and Future Research

This study has several limitations that need to be addressed in future research. First, although the cross-sectional design offers evidence of relationships between variables at a single point in time, it does not establish causal direction or temporal sequence. Furthermore, participant self-reports were the main method used to collect data. Although the possibility of common-method bias persists even with the more stringent unmeasured latent construct method. Consequently, future studies should adopt longitudinal tracking methods. Gathering data at various time points and from diverse sources (e.g., peer or mentor assessments) and applying cross-lagged models could yield stronger evidence to elucidate the causal relationships between variables.

Second, this study predominantly employed a simple random sampling method, and the proportion of students from Double First-Class universities in the sample is relatively high. Therefore, caution should be exercised when extending the results to non-Double First-Class universities. In addition, despite potential discrepancies in educational environments between disciplines, the study did not comprehensively report or compare sample distributions across different engineering subfields (such as mechanical, computer, and chemical engineering). Future research should thus employ stratified sampling to ensure representativeness across institutional levels and academic disciplines, evaluating the model’s generalizability across various subgroups.

Third, the research model concentrated on the distinct pathway of the macro-level variable “university organizational support,” leaving out important external environmental elements, including socioeconomic background, professor mentorship quality, laboratory atmosphere, and peer relationships. The results’ internal validity may be impacted by these factors’ interactions with institutional support. A comprehensive model that integrates macro-organizational, meso-process, and micro-individual elements should be developed in future research to systematically explain the generative ecology of engineering identity formation.

Furthermore, only 13.13% of the variance in engineering identity among post-graduates can be explained by this model, indicating that it only accounts for a subset of the factors influencing engineering identity. This demonstrates the intricate processes involved in graduate-level engineering identity development. According to social cognitive theory and related research, the development of professional identity among engineering students depends on factors including self-efficacy, interest, and learning engagement (Wu et al., 2021; Y. Li & Singh, 2022; Jones et al., 2013). As a result, future studies could integrate these variables into theoretical frameworks to investigate their potential mediating or moderating roles. Research findings in this field could be further enhanced and deepened by comparing and synthesizing these results with the mediating mechanisms analyzed in this study.

Finally, China’s unique “advisor-responsibility system” and its highly competitive academic environment serve as the foundation for this study. It is still to be determined whether these findings may be applied to individualistic cultures or alternative educational systems. More cross-cultural comparative research is required to verify the relevance of these insights in different educational settings and cultural contexts.

## 7. Conclusions

This study explores the relationship between university organizational support and identity among engineering postgraduates, as well as the mediating functions of major satisfaction and engineering belonging in the Chinese environment. The main findings in this research are as follows: (1) university organizational support is significantly and positively associated with engineering identity among engineering postgraduates; (2) major satisfaction mediates the relationship between university organizational support and engineering postgraduates’ engineering identity; (3) engineering belonging serves as a mediator connecting university organizational support to engineering identity among engineering postgraduates; (4) university organizational support and engineering postgraduates’ engineering identity are sequentially mediated by major satisfaction and engineering belonging. Based on these findings, this study suggests that universities should systematically develop a supportive graduate ecosystem by optimizing organizational resources. This can be achieved by enhancing resource allocation to improve major satisfaction and by fostering an inclusive, recognition-rich culture of engineering practice to strengthen students’ sense of belonging. These efforts, aimed at supporting students’ cognitive and affective engagement with their profession, are linked to the development of a more stable engineering identity.

## Figures and Tables

**Table 1:** Sample demographics (n = 939).

Variable		Number (n)	Percentage (%)
Gender	Male	563	59.96%
	Female	376	40.04%
Degree level	Master’s candidate	602	64.11%
	Doctoral candidate	337	35.89%
School type	Non-double first-class	358	38.13%
	Double first-class	581	61.87%
Academic ranking	Below 20%	116	12.35%
	20–40%	185	19.70%
	40–60%	340	36.21%
	60–80%	204	21.73%
	Above 80%	94	10.01%
Engineering practice experience	Non-practice-experienced	602	64.11%
	Practice-experienced	337	35.89%

**Table 2:** Summary statistics and correlation analysis (n = 939).

Variable	M	SD	1	2	3	4
1. Organizational support	3.09	0.68	1			
2. Major satisfaction	3.15	0.63	0.495 **	1		
3. Engineering belonging	3.18	0.65	0.405 **	0.431 **	1	
4. Engineering identity	3.19	0.69	0.327 **	0.403 **	0.405 **	1

Note: ** indicates *p* < 0.01.

**Table 3:** Mediating analysis results (n = 939).

Outcome Variable	Predictor Variables	R^2^	F	β	t
Major satisfaction	Constant	0.260	54.438 ***	1.61	10.41
	Gender			0.01	0.33
	Degree level			−0.09 *	−2.42
	School type			0.15 ***	3.93
	Academic ranking			0.02	1.34
	Engineering practice experience			−0.01	−0.22
	University organizational support			0.46 ***	17.19
Engineering belonging	Constant	0.239	41.799 ***	1.47	8.65
	Gender			−0.03	−0.68
	Degree level			−0.03	−0.69
	School type			0.07	1.82
	Academic ranking			−0.01	−0.82
	Engineering practice experience			0.01	0.38
	University organizational support			0.24 ***	7.70
	Major satisfaction			0.31 ***	9.00
Engineering identity	Constant	0.251	39.014 ***	1.31	7.07
	Gender			−0.09 *	−2.25
	Degree level			−0.12 *	−2.78
	School type			0.05	1.20
	Academic ranking			0.01	0.62
	Engineering practice experience			0.09 *	2.24
	University organizational support			0.10 ***	2.93
	Major satisfaction			0.25 ***	6.76
	Engineering belonging			0.27 ***	7.83

Note: * *p* < 0.05, *** *p* < 0.001.

**Table 4:** Path analysis results (n = 939).

	Effect	Boot SE	Boot LLCI	Boot ULCI	Effect Rate
Total effect	0.320	0.031	0.259	0.382	100%
Direct effect	0.101	0.035	0.033	0.169	31.53%
Total mediation effect	0.220	0.024	0.175	0.269	68.59%
University organizational support → Major satisfaction → Engineering identity	0.116	0.019	0.080	0.155	36.13%
University organizational support → Engineering belonging → Engineering identity	0.066	0.012	0.044	0.093	20.63%
University organizational support → Major satisfaction → Engineering belonging → Engineering identity	0.038	0.007	0.025	0.053	11.84%

Note: Boot SE: bootstrap standard error. Boot LLCI: bootstrap lower-limit confidence interval. Boot ULCI: bootstrap upper-limit confidence interval.

## Data Availability

The data materials presented in this study may be obtained from the corresponding author upon reasonable request.
